# Reflective Design in Action: A Collaborative Autoethnography of Faculty Learning Design

**DOI:** 10.1007/s11528-021-00679-5

**Published:** 2021-11-16

**Authors:** Shawn Bowers, Yu-Ling Chen, Yvette Clifton, Melissa Gamez, Heidi Hubbard Giffin, Meg Stanley Johnson, Laura Lohman, Linda Pastryk

**Affiliations:** Queens University of Charlotte, 1900 Selwyn Ave., Charlotte, NC 28274 USA

**Keywords:** Collaborative autoethnography, Course design, Design practice, Emotion, Iterative design, Faculty design, Learning design, Reflective practice

## Abstract

Research on how university faculty design courses has been limited and marked by modest detail on faculty design processes. Addressing this gap, seven faculty members supported by an educational developer at a teaching-intensive university used collaborative autoethnography (CAE) to explain how university faculty engage in reflective, iterative approaches to learning design. Collaborative analysis and interpretation of systematically collected data drawn from individual experiences in learning design reveal how faculty use reflection as a tool in learning design to recognize problems, devise solutions and constructively process emotions. Through reflection, faculty identify design solutions that are responsive to circumstances during course delivery, capture reasoning that informs design solutions for future course iterations and accurately gauge the appropriate timing of design changes based on factors such as scale and feasibility. This article offers detailed ethnographic evidence and new findings that enrich our understanding of claims made in previous interview-based studies of faculty design.

As highlighted in the February 2021 special issue of *Educational Technology Research and Development* “Shifting to Digital,” limited published research addresses how university faculty design courses. Responses in the issue to Bennett et al. (2017) reiterated the scant research on faculty practice in learning design. Studies have often relied on isolated interviews to gather data, offered limited detail on faculty design processes and incorporated little faculty voice (Baldwin et al., 2018; Lohman, 2021). Collaborative autoethnography (CAE) is ideal for addressing this gap to clarify how faculty approach learning design. CAE engages researchers as research participants over an extended time, combines individual data collection with group meaning-making to understand individuals’ experiences in a community and incorporates multiple researcher-participants’ voices (Chang et al., 2013).

In this research article, seven faculty members supported by an educational developer at a teaching-intensive university use CAE to explain how faculty engage in reflective, iterative approaches to learning design in university courses. Collaborative analysis and interpretation of systematically collected data drawn from individuals’ experiences in learning design reveal how faculty use reflection as a tool in learning design to recognize problems, devise solutions and constructively process emotions. Through reflection, faculty identify design solutions that are responsive to circumstances during course delivery, capture reasoning that informs design solutions for future course iterations, and accurately gauge the appropriate timing of design changes based on factors such as scale and feasibility. Detailed ethnographic evidence provides new insight on faculty learning design, including the role of emotion and mid-semester reflection-driven design changes, while adding new data to support claims made in previous interview-based studies of faculty design.

## Literature Review

### Faculty Design

This CAE case study in faculty learning design contributes to modest literature documenting faculty design practice, which, as Baldwin et al. (2018) noted, has been outweighed by prescriptive guidance on and models of design. Noteworthy studies of faculty design practice have used one-time interviews with faculty to gather data. Interviewing 30 faculty from 16 Australian universities with experience in face-to-face and online instruction and 14 instructors from urban public four-year colleges and universities with experience designing online courses, Bennett et al. (2017) and Baldwin et al. (2018) developed descriptive models of faculty design processes.

Bennett et al. (2017) identified commonalities in faculty design processes across Australian institutional contexts and disciplines. Faculty designing a new course began by focusing on learning outcomes or content. Faculty redesigning a course used varied starting points based on their conceptualization of the design problem and specific changes needed. Interviewees typically established an overall course framework and then shifted to detailed considerations such as selecting readings, developing learning activities, and determining assessments of learning. Faculty did not follow a systematic or linear sequence of steps (Bennett et al., 2017).

Baldwin et al.’s (2018) interviews with faculty who design online courses highlighted several points of intersection with Bennett et al.’s study. Like the Australian faculty, Baldwin et al.’s interviewees, drawn from unspecified national context(s), infrequently used formal instructional design models. Baldwin et al. developed a descriptive process model illustrating how faculty design begins with determining learning objectives and resources and continues with structuring and chunking content, determining if the learning management system can accommodate the design, delivering the course and then using end-of-semester student evaluations to validate or modify the design; onto this model Baldwin et al. superimposed ADDIE.

Several researchers have highlighted the iterative and continuous nature of faculty design (Beetham & Sharpe, 2013; Bennett et al., 2017). Sharpe and Oliver (2013) noted that most faculty design activities are redesign, or improving a course through iteration, rather than initial design. Faculty design before, during and after a delivery of the course, adapting initial designs during delivery and reflecting after course delivery to identify possible improvements for the next course iteration (Bennett et al., 2017). Reasons for faculty course redesign efforts include feedback from students and colleagues, updating content, addressing problems noted while teaching, incorporation of online components and changes in instructional staffing (Bennett et al., 2017).

CAE can extend research on faculty learning design in several ways. First, while one-time interviews prompting faculty to recall complex processes have offered limited detail, CAE can enable consideration of micro-level design decisions (Bennett et al., 2017). Second, Bennett et al. (2017) stressed as important for future research faculty records of their design activities and analysis of faculty language describing their design processes, which CAE enables. Finally, the traditional researcher-participant distinction has prompted the incorporation of limited faculty voice in relation to researchers’ voices (Baldwin et al., 2018; Bennett et al., 2011; Bennett et al., 2017). CAE is an ideal method for incorporating faculty voice.

### Reflective Design

The title, “Reflective Design in Action,” captures reflective design changes that faculty contemplate during course delivery, which previous research has not explored in depth. The title also echoes noteworthy literature on reflection, particularly the contributions of Schön. While Schön’s and other scholars’ work on reflective design did not drive this CAE project, shape data collection or inform data analysis, their work provides an important context in which our findings can be situated.

Schön (1983) addressed reflective practice as a way that professionals gain awareness of their tacit knowledge and learn from their experience through reflection on action and reflection in action. Reflection on action occurs after action, whereas reflection in action is concurrent with action. Through reflection in action, one takes an action and uses its effect as feedback to inform decisions on how to continue or modify one’s approach and sustain an ongoing conversation with the larger situation to inform future actions. Schön’s conceptualization of reflection was intimately connected with design decision-making, as seen in his treatment of architectural design as an example of “design as a reflective conversation with the situation” (1983, p. 76).

Schön’s work shaped two conceptual models of reflection in design intended to support student designers and their instructors. Hong and Choi (2011) used literature on reflection to create a conceptual model of reflective thinking in a design process and help educators and instructional designers provide learning environments that promote novices’ reflection when solving design problems. Their three-dimensional model delineated timing, objects and levels of reflection. Applying this model, Hong and Choi (2019) used survey data from a biomedical systems and devices course to determine student designers’ reflection patterns and differences between high- and low-performers’ reflection patterns. Also focusing on reflection by those learning design, Kavousi (2017) developed the Metacognitive Design Thinking Framework, which consists of reflective process knowledge, reflective process monitoring and reflective process control. Kavousi et al. (2020) gathered qualitative data from students in a first-year design lab to identify patterns in their metacognitive thinking, relate them to high- and low-quality designs and explore how metacognitive thinking and actions influence students’ design learning.

Faculty reflection in learning design contexts has received less attention. Jung et al. (2021) used reflective journaling by five faculty members during seven weeks of teaching online courses during the COVID-19 pandemic to examine emergency online teaching experiences. Their analysis explored the problems encountered, experiences and resources used to solve problems, faculty actions during emergency online teaching, how faculty reflected on their actions and emergency online teaching and what differences occurred in faculty members’ experiences over time. Findings included the relative prevalence of student-related, technology-related, content-related, and time-management problems. To address problems in emergency online teaching, faculty drew on previous teaching experience and student suggestions and applied pedagogical strategies, technical solutions and logistical arrangements. While the study does not explicitly address how faculty members’ reflections relate to learning design, it underscores the value of CAE for exploring reflection in faculty learning design.

## Methods and Context

### Collaborative Autoethnography

CAE is a qualitative research method in which research teams collect autobiographical data and collectively analyze and interpret it to understand sociocultural phenomena such as mothering, study abroad and workplace emotion (Anderson et al., 2020b; Chang et al., 2013; Garbati & Rothschild, 2016; Lapadat, 2017). CAE team members leverage their own experience as data sources and are both researchers and research participants. CAE often unfolds through alternating individual and group work, with individual researcher-participants’ data probed through conversation. Collaboration helps team members uncover assumptions, explore alternative interpretations and bring varying disciplinary perspectives to data (Chang et al., 2013; Geist-Martin et al., 2010). CAE teams range from two to ten members and may include a non-autoethnographer who supports other members (Chang et al., 2013).

CAE teams conduct data collection, analysis, interpretation and writing in varied ways. Individual team members may contribute past or present autobiographic data through memories, self-observation, self-analysis, reflection and responses to shared prompts. Teams adopting an analytical approach commonly use emergent codes to identify key themes and consider what data means in context (Anderson et al., 2020b; Chang, 2013; Garbati & Rothschild, 2016), often using individual journaling, discussion and collaborative writing in iterative cycles (Anderson et al., 2020a; Vans Katwyk & Seko, 2017). Scholars have used CAE as both a research method to examine experiences in within higher education (Gates et al., 2020; Roy & Uekusa, 2020) and an “authentic learning activity” (Lee, 2020).

### Case Study Context and Methods

This case study focuses on reflective, iterative design practices of faculty in a small, private teaching-intensive university in the United States. Seven faculty members, supported by an educational developer with faculty rank, engaged in concurrent individual data collection through journaling about their experiences in learning design throughout one 15-week semester, followed by group discussion and interpretation of shared data through inductive qualitative coding. The educational developer provided support through project management, engagement with secondary literature and methodology. The eight CAE team members spanned disciplines in humanities, arts, and natural sciences and included full-time and adjunct faculty. In addition to team members’ previous teaching experience at the university and K-12 levels, six of the seven faculty members brought experience in learning design gained through participation in a four-week Course Design Institute that included application of systematic and backwards design with emphasis on significant learning (Dick et al., 2015; Fink, 2013; Lohman, 2019; Wiggins & McTighe, 2005). The five who participated in the modified Course Design Institute during the COVID-19 pandemic also addressed digital accessibility and gained experience in the selection of instructional technologies from a learning design perspective (Lohman, 2020).

Consistent with CAE and the common faculty practice of recording notes about issues to change or develop in the future (Bennett et al., 2017), team members were prompted to journal at least twice per week on one course they were teaching that they anticipated teaching again. The prompt invited members to use a journal format and media of their choice and include varied content such as descriptions, observations, connections to other experiences, ideas, questions, feelings and other reflections. Two members noted already having such a practice through handwritten notes following class sessions or digital additions to a syllabus file. Others noted regularly making but not writing such observations and reflections. A significant difference in the reflecting practice therefore was undertaking it as a coordinated project in response to a shared prompt to record members’ observations and reflections within the CAE methodology.

After the semester, the seven faculty members shared selected journal entries and commentary on them as data for collaborative analysis, discussion, and interpretation. Data contributions were shaped by three mutually agreed-upon prompts: share 1) three representative entries, accompanied by explanations of their representativeness; 2) two entries illustrating how reflection processes led to a change in approach to the target course during delivery in the semester of journaling, accompanied by an explanation and 3) two or three entries illustrating how reflection processes led to an anticipated change in approach to the target course after the semester of journaling, accompanied by an explanation. The educational developer gathered submissions and then shared them with all team members to prevent peer influence on selection. With the semester of course delivery taken as the unit of action, submissions illustrated Schön’s reflection in action even when containing some changes flagged for later implementation.

The seven faculty members individually coded the representative submissions using emergent qualitative coding. Four and three coders used process coding and emotion coding, respectively, incorporating In Vivo codes. Process coding uses gerunds to identify actions (e.g., hitting, thinking), while emotion coding identifies emotions (e.g., anger, elation) to explore participants’ interpersonal and/or intrapersonal experiences. Emotion coding is suitable when examining phenomena involving social relationships, reasoning, decision-making and judgment (Saldaña, 2021). Process coding and emotion coding by multiple coders were digitally merged into two files; coders in each subgroup intensively discussed and reached consensus where possible. This approach honored CAE emphasis on group meaning-making through discussion and guidance against quantifying interrater reliability when working with relatively unstructured data (Morse, 1997; Saldaña, 2021). The seven faculty members used insights gained through coding representative submissions to interpret submissions to the second and third prompts. Quotations from journal entries were used with faculty consent and anonymized; bracketed interpolations were used in reporting findings as needed to maintain compliance with ethical standards.

## Findings

How do faculty engage in reflective, iterative approaches to learning design in university courses? This CAE case study revealed that faculty use continuous reflection to recognize problems, devise design solutions and process emotions constructively in support of such design solutions and in pursuit of more positive learning outcomes. Adapting to current circumstances including student responses, they identify specific opportunities to make learning design changes both while the course is in progress and between course iterations, identifying the appropriate timing of each change based on factors such as scale and feasibility. In this study to date, circumstances included a largely HyFlex semester implemented with relatively short notice in response to COVID-19 pandemic conditions to prioritize safety and serve all students. Different from both hybrid and HyFlex modalities as understood pre-pandemic, this large-scale, rapid implementation of HyFlex was termed hybrid and multi-access at the university and is designated here through “hybrid.” Faculty members teaching “hybrid” courses were expected to provide instruction in-person, remotely and online in a single course, at times simultaneously, as needed to support students. Faculty members approached this semester with a mixture of feelings and a range of attitudes. While one noted in an early journal entry that it was “hard to stay motivated,” another “looked forward to the challenge.” Overall, faculty members reported feeling both cautious and hopeful.

### Representative Reflections

The week-to-week reality of this semester clearly revealed itself in the researcher-participants’ representative journal entries. Process coding clarified that reactions to student engagement, attendance and participation predominated, along with the personal reactions of the faculty whose strong desire was for students to learn and demonstrate that learning. While the first and largest takeaway from the data seemed to be an overwhelming expression of frustration, a deeper dive into the processes underlying those raw expressions revealed an extensive list of codes that fell into six categories.

The categories used to group process codes were Student, Faculty/Classroom/Teaching, Pedagogy, Emotional Processing, Cognitive Processing and Journaling (Table 1). Numerous codes emerged from the representative entries, with predominant codes including desiring, questioning, adapting/adopting, rationalizing/valuing, feeling and acknowledging/addressing. While each entry was unique, the researcher-participants-turned-process coders were surprised to recognize similarities in their thought patterns and processes. Themes included frustration, reflection and compassion. For example, faculty were frustrated with student engagement that was exacerbated by the “hybrid” modality. Faculty reflected on their struggles through questioning their current practice and how to make change, and they showed compassion to themselves and their students despite the challenging situation by acknowledging limitations.

**Table 1:** Process codes and categories accompanied by objects and descriptors of processes

CATEGORY	Student	Faculty/Classroom/Teaching	Pedagogy	Emotional	Cognitive	Journaling
CODE • Descriptors or objects of the process	ADJUSTING • To modality COLLABORATING CONTRIBUTING • To class ENGAGING • With class EXPRESSING / SHOWING • Boredom • Uninspired • Enjoyment HELPING • Classmates RESPONDING • To questions	APOLOGIZING • To students COMMISERATING • With colleagues COMMUNICATING • With students DESIRING • Student contribution • Small groups • Learning • Meaningful impact • Connection • Student independent thinking • Streamlined approach FORGETTING • Assignment LEARNING • From students REPEATING • Information SECOND-GUESSING • Classroom practices • Successes • Student Assertions • Self • Accessibility to content • Decision making STRUGGLING • Engagement • Technology • LMS • Modality • Student feedback • Workload	ACCOMMODATING • Students • Classroom protocols ADAPTING / ADOPTING • Focus • Expectations • Planning • Needs • To modality • To technology • Alternative methods • New mindset • Flexibility CREATING / DESIGNING • Connections • Accountability DEVELOPING • In-class activities PROVIDING • Instructional support • Meaningful experiences RATIONALIZING / VALUING • Course content • Course impact • Course goals, objectives • Approach, pedagogy • Process • Circumstance • Methodology REDUCING • Penalties	APPRECIATING • Students • Student adaptability EMPATHIZING • With students • With self FEELING • Frustrated • Disappointed • Overwhelmed • Nervous, apprehensive • Scattered, distracted • Failing students • Offended • Exhausted • Insecure • Defeated • Confident • Hopeful • Excitement • Stressed • Optimistic • Not prepared • Compared • Defensive • Sad • Self-conscious • Surprised NEEDING • A break	ACCEPTING • Limitations • Circumstances ACKNOWLEDGING / ADDRESSING • Limitations • Value • Hybrid challenges • Technology challenges • Students struggling • Student efforts • Challenges, issues • Successes, advantages • Own strengths • Workload • Feelings • Emotions • Class community • Need for change • Time constraints • Lack of engagement • Solutions • Different perspectives ASSESSING/ EVALUATING • Course • Content • Past experiences • Engagement • Feedback • Successes • Emotions • Methodologies • Future • Teaching • Self • Others • Options DISCOVERING / OBSERVING • Level of engagement • Challenges, issues • Confusion • Student characteristics • Solutions • Findings QUESTIONING • Workload • Actions • Decisions • Teaching • Serving students appropriately • Connections • Student intent • Self • Meaningfulness • Learning • Influence of modality • Students	ANALYZING • Journal content APPRECIATING • Reflecting CAPTURING • Raw emotions • Thoughts CHANGING • Voice COMPARING • Journaling techniques –narrative vs annotated EXPLAINING • Style • Process REFLECTING • Journaling • Style WRITING • Narratives

**Fig. 1:**
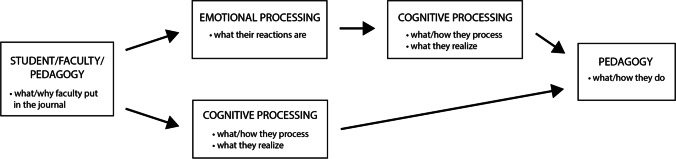
Summary of key cause-and-effect or sequential connections in representative entries identified through process coding

Analysis of the data also revealed connections between processes. Such cause-and-effect or sequential connections in process codes (Saldaña, 2021) occurred across and within categories. For example, spanning two categories, students not engaging triggered faculty feeling frustrated. Within the category of cognitive processing, faculty acknowledging issues frequently led to their addressing issues. Common cause-and-effect or sequential connections are summarized in Fig. 1. In representative entries, which were predominantly about challenges and issues, faculty expressed emotions in response to the issues. Following the emotional processing, faculty used cognitive processing to evaluate their current practice and contemplate options to improve the situation by making a change. Some issues spurred cognitive processing without emotional processing. During cognitive processing faculty also realized limitations and compassion. Some cognitive processing led to actions of modifying or adjusting pedagogy.

Insights from process coding about the role of emotional processing in guiding learning design decisions were deepened through other team members’ independent use of emotion coding. While emotion has not been prominent in prior research on faculty learning design, analysis of representative entries using emotion coding clarified how emotions were significant factors guiding course design. When journaling, researcher-participants noted their emotions, labelled them, reflected on them and avoided becoming punitive. For example, one faculty member noted “there is a theme of overwhelm and feeling like I am failing my students...I second-guessed all of my decisions.” Another professor acknowledged that she was “feeling a bit insecure” one day but “felt confident with my examples and preparedness” another day. Emotive desire for positive learning outcomes demonstrated that faculty used journaling not only for reflection, but also as a pedagogical tool: they followed their emotions as constructive stimuli for targeting issues and reconsidering class strategies.

Coders placed emotion codes into seven categories: Fulfillment, Rational, Determination, Exhaustion, Sadness, Anger and Fear (Table 2). Representative journal entries evidenced an abiding sense of Determination that was a significant impetus for researcher-participants’ choices in learning design, even during periods of Exhaustion. The positive emotional range of Determination codes suggests that faculty understood that course design elements could be changed. Fulfillment was the category with the largest number of codes used. Coupled with Determination, Fulfillment codes suggest that faculty generally processed negative emotions such as exhaustion, overwhelm and anger through rationalizing them. Sadness, Anger and Exhaustion were the categories with the smallest number of codes used.

**Table 2:** Codebook of emotion categories with data samples and Representative codes

Categories	Code descriptions and data samples	Representative codes
Fulfillment	Codes refer to faculty members’ genuine feelings of joy, connection, curiosity, satisfaction, and self-acceptance. These past-oriented codes represent feelings in the present as a result of past actions. *“Hands on is definitely helpful and* *engaging* *– I liked the way it spurred conversation …*.”	Acceptance Happy “I love” Self-assurance “So appreciative” “Wow! What a great surprise”
Rational	Codes refer to faculty members’ emotive factors in practical and theoretical reasoning: self-awareness of both negative and positive emotions. *“The ask to pivot the research papers to w.a. was too nuanced.* *I don’t think I did a good enough job* *explaining how the context and audience changed.”*	“Affirmed” Anticipation “I don’t think I did a good enough job” “Is a bit harder” “It just wasn’t happenin’” Self-aware “Truth-be-told”
Determination	Codes refer to faculty members’ feelings of desire, optimism, and confidence. These future-oriented codes represent feelings in the present in anticipation of future results. *“I* *want* *students to experience this class as holding real potential for their skill sets – conceptual analysis skills and visual analysis skills – a whole brain experience as research confirms – and leave the course thinking that significant learning occurred here – that they thought about what they were visually absorbing and physically connecting with in their material environments – as they moved daily throughout their environments and how this was meaningful for them.”*	Confidence Hope “I can make this work” “Need to” “Want”
Exhaustion	Codes refer to faculty members’ feelings of fatigue, sense of resignation, and being overwhelmed. *“I continue to* *struggle* *with making our hybrid in-person sessions engaging, but the students are not doing the work to help carry this discussion and activities …. Many of them haven’t even purchased the books, ten weeks into class.”*	“Exhausted” Fatigue “I need a break” “Low energy” “Mentally drained” Overwhelmed “Struggle”
Sadness	Codes refer to faculty members’ feelings of discouragement, disappointment, and disillusionment. *“I feel* *let down* *when students do not come to class and provide no reason – or take a ‘personal day’ when we are planning a speaker...”*	Disappointment Dismay “Let down” Pain “Sad”
Anger	Codes refer to faculty members’ feelings of alarm and frustration*.* *“It was* *frustrating* *for the students online because of the sound quality, and it was* *frustrating* *to me because it was harder to interact with the whole class. The ultimate* *frustration* *was that this issue could only be tolerated but not solved.”*	“Anger” Annoyance Frustration “Hate” “Having a hard time” “Over-reacted”
Fear	Codes refer to faculty members’ feelings of uncertainty and anxiousness. *“... feeling like I am* *failing my students* *throughout the semester’s entries. I second-guessed all my decisions, from choosing hybrid* vs. *online to which assignments to keep and modify to what the hybrid classroom should even look like.”*	Anxiety “Failing my students” “Feeling a bit insecure” Hesitancy “Nervous” “Not sure” Uncertainty

Quotation marks around codes designate In Vivo Codes

While negative emotions, such as anger or fatigue, were consistently coded in representative entries, they did not outweigh positive emotions, such as satisfaction and determination. Often, exhaustion and fear arose from the unpredictability of the “hybrid” modality, along with expected accommodations for unique student circumstances prompted by pandemic conditions. Exhaustion, then, can be understood as an extenuating factor. Negative emotions, when reflected upon, alerted faculty to potential failures in the course, unless they were constructively remedied. The only negative emotion that frequently occurred was fear. Fear catalyzed faculty to note potential design changes—fear of failure to accomplish basic course goals, fear of losing control of the class, fear of comparison with other faculty, fear of course evaluations and fear of student complaints: “Very frustrated when receiving email about clarity about deadline. I felt I have been working really hard to the point that I don’t agree with but because one of my colleagues is super clear, I don’t want the students to complain through comparison.”

Representative entries captured intrapersonal and interpersonal emotional dimensions of class experience. Intrapersonal entries revealed initial states of vulnerability, such as anger, fear and discouragement. Strategic attention to concerns generating these emotions yielded constructive solutions and feelings of determination, self-assuredness and satisfaction. Interpersonal entries generally demonstrated self-regulation of emotions to advance course goals. Faculty vulnerability with students included seeking student approval, expressing frustration with student initiative, displaying sympathy, empathizing with students’ emotions, and extending compassion: “I intentionally do not hand out new assignments during this time because I know the students have so much work to do.”

### Within-Semester Change

Process coders observed that reflections capturing within-semester change were less emotional and more action-based than the representative entries as faculty recognized issues and limitations in their courses and created varied solutions to address them. The overwhelming sense of frustration noted through process coding of representative reflections was not as dominant in these “within-semester change” reflections as faculty found ways to address and minimize challenges they were facing in their courses. A lack of student engagement due to better weather, no spring break, and the “hybrid” modality drove faculty to redesign aspects of their courses during the semester. For example, faculty changed learning activities to foster engagement and discussion: “the students are not reading, so they don’t have anything to talk about. By adding the discussion board prompts, there is an incentive to read and we can always use the posts to jumpstart a conversation in class if no one wants to speak up.”

Another leading factor prompting faculty to consider within-semester design changes was students’ inability to understand or process course content and information because of the “hybrid” modality and not being in person. One faculty member noted that online students often had both webcams off and microphones muted during videoconference class sessions. To accommodate this user group, the faculty member modified the class to “lecture for first 15-20 minutes, then students write for the papers or [Integrative Assignment] and those at home are free to go or hang out so I can answer questions.” Otherwise, the faculty member noted, “I feel as though I am ignoring one set.” This excerpt illustrates faculty members’ use of a flexible mindset and unique pedagogical approaches to address challenges faced by students and accommodate multiple simultaneous modalities.

When considering within-semester design changes, faculty also utilized practices they had not used in previous course iterations in different modalities. One faculty member noted, “I had been thinking about how to increase the effectiveness of teaching in a hybrid format since half of my students went online and half of the students were in-person simultaneously... I supplemented information through follow-up announcements.” Such initiatives demonstrate faculty members’ commitment to their students and willingness to address challenges and not allow them to dominate their courses. Though not all faculty were confident that their methodologies were effective, six of the seven faculty implemented design changes in their courses during the semester.

While process coders observed that these reflections were less emotional and more action-based than the representative entries, emotions still played a role and patterns in contributing emotions were detected. Course design changes made within the semester were largely the result of negative emotions. The challenging unknowns of “hybrid” teaching and attendant issues of content delivery with low student engagement required prompt responses. This “hybrid” format prompted frustration across researcher-participants: “This hybrid modality is not serving anyone! It’s too hard to deploy two different pedagogies simultaneously.” The difficulty was not in how to adapt to meet specific student needs mitigated by the unique circumstances of the pandemic, but rather points to a taxing process for faculty that led to emotional exhaustion. Avoiding some issues related to the “hybrid” modality, one faculty member had intentionally planned a fully asynchronous online class, with the intent not to adjust course design during the semester. For another instructor who began the semester in the “hybrid” modality, the lack of student initiative and attendance resulted in frustration and anger. Processing these emotions through journaling, she decided to shift the focus to asynchronous learning resources for the last two weeks of the semester. This researcher-participant felt satisfied with the decision: “I did meet my students where they were,” providing “quality instruction to those who wanted it. Good instruction can happen in person or virtually, and by focusing on those who didn’t want to be in the classroom, I was effectively short changing [*sic*] everyone.”

Unable to pivot and plan with some certainty as in previous semesters of in-person modality, the faculty could not anticipate outcomes as effectively in the “hybrid” modality: “Being not able to anticipate this situation, I experienced some trial and error when trying to make modification along the way. This change came from remembering past successes.” As faculty attempted to adapt the content delivery of the in-person classroom to the “hybrid” modality, issues surfaced with the ability of the technology to deliver a parallel experience. One instructor was forced to find ways to accommodate for the shortcomings of the technology. Likewise, another faculty member highlighted this desire to create a rewarding experience that mimicked previous in-person experiences: “I am aiming for a class experience which provides a material sense of being there.”

Instructors self-regulated negative emotional dynamics and demonstrated both a desire and determination to make within-semester modifications to ensure a quality learning environment. Negative emotional states were processed constructively to bring about positive outcomes. Faculty noted varied levels of emotional satisfaction from low to high with their mid-course design solutions and in some cases, noted positive student feedback to course adjustments. For example, one faculty member implemented student accountability partners for end-of-semester goals partway through the semester and later reflected, “it turned out to be the most effective thing I did all semester and students reported they wished we had done this the whole semester.” One faculty member noted lingering dissatisfaction: “I’m not happy with the solution, but I could not think of anything better.” Generally, by attending to their emotional states related to their teaching, instructors strove to effectuate positive course outcomes within the semester.

### Between-Semester Change

During the semester, all seven faculty members recorded reflections identifying specific design changes they wished to make in the next iteration of their target courses. Process coders observed that faculty between-semester course design changes continued to be student-driven. In contrast to within-semester change reflections, where faculty often made efforts to address student engagement, in between-semester change reflections, faculty focused on fundamental needs of students concerning what they learned, how they learn, and equality. Changes faculty noted for future iterations included course delivery, course materials, learning objectives, assignments and grading. Overall, these entries were more specific and directed and less focused on cognitive and emotional processing prevalent in the representative reflections. Many faculty members could identify the solution without much contemplation and were ready or eager to implement it in the future. Processes such as recognizing, acknowledging, evaluating, considering and changing were prevalent.

Topics addressed in these between-semester change reflections required a long view of the whole semester and reflected bigger-picture thinking about the effectiveness of assignments, assessment methods and grading. Some issues of pacing and scheduling could not be fully addressed until after the semester: “They had a lot this week: the exam, reflection, memoirs, and Integrative Assignment due. It was too much. Too much for me to grade and too much for them to do. I did not want to take any of these assignments out, but I could definitely adjust/space them out differently.” Such reflections capture how faculty members recognized issues in and negative consequences of learning design decisions and identified the most feasible time to make needed changes. Additionally, some possible course changes required consultation with other faculty, such as changes in learning community assignments that affect other faculty members or review of curricular learning objectives that need to be considered by all faculty in a particular program. As one faculty member noted, “I think this is a place where I need clearer connections with how it fits into our curriculum. There are concepts that I think we should just be reviewing, but they seem new to students - need to make sure whether I should be teaching a skill or reviewing a skill.”

During the semester, faculty were limited in their ability to introduce additions to the course, such as new assignments and new course materials, but they made plans to introduce them in future semesters. In reflections capturing intentions for between-semester change, the “hybrid” modality and issues surrounding the pandemic were not as predominant as in the representative and within-semester change reflections, but they did seem to influence some of the faculty considerations for future changes. Overall, these reflections contained a definite sense of moving forward and hopefulness, with a predominant focus on improvement of course delivery to best meet the needs of all students.

Emotion coding of representative reflections lends greater insight into the emotional states driving such between-semester design changes. As faculty anticipated a future iteration of their courses, emotional states that surfaced most often were in the categories Determination, Rational processing and Fulfillment. Researcher participants were determined and hopeful of achieving a quality learning environment in their future course iteration: “Next term, I will implement this tool, and am curious to see its impact from both the students’ perspective... and my perspective. It’s funny how a little change has the potential of packing a big punch!” Other faculty captured their reasoning but not a specific decision about how they would proceed in the next iteration: “Letting the students talk through what they learned from the readings by making a Flipgrid video was a new strategy I implemented... to provide another modality... for diverse learners. Because it was new, I underestimated the time I needed for grading... [which] led to less timely grading and made me re-evaluate the effects of this assignment.” In one instance, expressions of anger at low student performance prompted the rethinking of assessments for the next course iteration: “This is a skill building class that lays a foundation for students... this semester showed that I need to be putting more emphasis on their effort and not just completion.”

As reflected in such quotations, researcher-participants expressed an emotional range of concern for student workload and initiative and for their own course goals and pacing of content, prompting a reevaluating of future course design. As emotions fueled reflections that were attuned to “problems” needing attention, there were issues that could not be easily or promptly repaired within the semester, such as assessment, grading, course pacing, scaffolding and educational technology tools. These were then flagged as specific concerns to address in the next course iteration.

## Discussion

This CAE study contributes new findings and detailed data to previous literature on faculty practice in learning design. First, it clarifies processes through which faculty make within-semester changes to learning designs, changes that have been overshadowed in previous literature by attention to post-semester reflections and design changes (Baldwin et al., 2018; Bennett et al., 2017). It demonstrates that students’ responses are not only a significant impetus for course design changes *after* a semester (Baldwin et al., 2018) but also for within-semester changes. Second, CAE findings expand on modest prior observations on emotion in faculty design, such as instructor frustration with uploading course materials to learning management systems and eagerness for student end-of-semester feedback (Baldwin et al., 2018). This CAE study exposes the significant and constructive role that emotion can play in learning design when faculty have responsibilities for course design, along with course development and delivery. Finally, the recurring student orientation of faculty reflections suggests that a “human-centered approach” to learning design is implemented by faculty in some settings (Karakaya, 2021).

This CAE study also provides additional data and faculty perspective underscoring findings in previous literature. It confirms the importance of unpredictable and contingent aspects of learning and faculty responsiveness to that reality, here prompted by the large-scale “hybrid” modality and accommodations of student needs (Beetham & Sharpe, 2013). Faculty members’ decisions to change learning activities mid-semester and flag larger-scale design changes for a future iteration reinforce the value of a layered understanding of “design for learning” that ranges from the course to the single learning activity (Beetham & Sharpe, 2013; Boyle, 2010). Faculty use of their previous in-person teaching experience as a reference point in their reflections underscores the importance of designers’ prior experience and tacit knowledge (Sharpe & Oliver, 2013). Finally, findings support Sharpe and Oliver’s (2013) observation that faculty take a predominantly pragmatic approach to design focused on addressing specific problems rather than following a design model.

The strong orientation of faculty design in this CAE study away from design models and towards pragmatic design to solve problems reinforces Beetham and Sharpe’s elaboration of design as “praxis,” particularly “in the widely used sense of iterative, reflexive professional learning” (p. 53). Reflecting provided a learning opportunity, especially in challenging circumstances. For the seven faculty members, the journaling of their experiences, often while teaching individual courses simultaneously in both in-person and online modalities, proved a worthwhile exercise for improving course design during a semester and for a future semester. The consistent attention to their emotions demonstrated the value of emotions as signifiers of issues and remedies. Looking ahead to a future semester, they felt concern, determination and hope for achieving a quality learning environment and identified new design solutions. Faculty writing their reflections captured the potential of design as a source of learning for designers, in addition to supporting learning for learners. As such, this study underscores European research supporting faculty reflection on their design practice as a form of professional development (Wasson & Kirschner, 2020).

## Limitations and Suggestions for Future Research

Along with rich detail afforded by the CAE methodology, this case study also carries limitations. To date, this study is based on one semester of reflective writing, carried out during pandemic conditions. Sustained data collection and analysis are needed to clarify the representativeness of findings. In particular, more research on the role of emotions and designers’ attention to emotions is warranted. Affective processing has garnered a place in models of reflective thinking in a design process, but it has been reported as relatively uncommon among novice designers in relation to faculty, especially those engaged in teaching during the pandemic (Hong & Choi, 2011; Hong & Choi, 2019; Jung et al., 2021; Kavousi et al., 2020). In addition, a small, teaching-intensive institution may not be representative of other institutional settings; future research may address faculty course design practices in larger institutions with varied missions. While the researcher-participants are ethnically and racially diverse, gender diversity was not captured in this study and may be addressed in future research. Finally, the potential impact of journaling or writing one’s reflections, as opposed to simply reflecting, cannot be isolated in this CAE case study.

## Conclusion

As a case study in faculty learning design, this article both sustains and adds new findings to previous literature on faculty design practice. Faculty use continuous reflection to recognize problems, devise iterative design solutions and process emotions constructively. Through such reflection, they identify design solutions that are responsive to current circumstances during course delivery, capture reasoning that informs design solutions that are best suited for a future iteration, and accurately gauge the appropriate timing of design changes based on factors such as scale and feasibility. Emotion can play a more prominent and constructive role in such learning design decisions than suggested by previous research, and a student-focused approach often drives design decisions.

The qualitative detail afforded by ethnographic methods such as CAE also offers valuable insights for learning designers and administrators, including those who regularly work with, lead, and support faculty in course design. Common ground, such as interest in student engagement and pragmatic and contextual approaches to design (Stefaniak, 2021), can assist communications and mutual understanding between faculty and professional learning designers who are often separated through organizational structures, organizational culture, and discourse that positions faculty as subject matter experts and instructional designers as learning experts. Broadly applicable, CAE is also a valuable research method for understanding how learning design is practiced by a wide range of such professionals within higher education, K-12, military, and corporate settings.
